# Randomized clinical trial of a phytotherapic compound containing *Pimpinella anisum*, *Foeniculum vulgare*, *Sambucus nigra*, and *Cassia augustifolia *for chronic constipation

**DOI:** 10.1186/1472-6882-10-17

**Published:** 2010-04-30

**Authors:** Paulo D Picon, Rafael V Picon, Andry F Costa, Guilherme B Sander, Karine M Amaral, Ana L Aboy, Amélia T Henriques

**Affiliations:** 1 Department of Internal Medicine and Chief of the Clinical Research Unit at Hospital de Clínicas, Universidade Federal do Rio Grande do Sul, Porto Alegre, Brazil; 2 Clinical Research Unit, Hospital de Clínicas, Universidade Federal do Rio Grande do Sul, Porto Alegre, Brazil; 3 Faculty of Pharmacy, Universidade Federal do Rio Grande do Sul, Porto Alegre, Brazil

## Abstract

**Background:**

A phytotherapic compound containing *Pimpinella anisum *L., *Foeniculum vulgare *Miller, *Sambucus nigra *L., and Cassia *augustifolia *is largely used in Brazil for the treatment of constipation. However, the laxative efficacy of the compound has never been tested in a randomized clinical trial. The aim of this study was to evaluate the efficacy and safety of the product.

**Methods:**

This randomized, crossover, placebo-controlled, single-blinded trial included 20 patients presenting with chronic constipation according to the criteria of the American Association of Gastroenterology. The order of treatments was counterbalanced across subjects: half of the subjects received the phytotherapic compound for a 5-day period, whereas the other half received placebo for the same period. Both treatment periods were separated by a 9-day washout period followed by the reverse treatment for another 5-day period. The primary endpoint was colonic transit time (CTT), measured radiologically. Secondary endpoints included number of evacuations per day, perception of bowel function, adverse effects, and quality of life.

**Results:**

Mean CTT assessed by X ray was 15.7 hours (95%CI 11.1-20.2) in the active treatment period and 42.3 hours (95%CI 33.5-51.1) during the placebo treatment (p < 0.001). Number of evacuations per day increased during the use of active tea; significant differences were observed as of the second day of treatment (p < 0.001). Patient perception of bowel function was improved (p < 0.01), but quality of life did not show significant differences among the study periods. Except for a small reduction in serum potassium levels during the active treatment, no significant differences were observed in terms of adverse effects throughout the study period.

**Conclusions:**

The findings of this randomized controlled trial allow to conclude that the phytotherapic compound assessed has laxative efficacy and is a safe alternative option for the treatment of constipation.

**Trial registration:**

ClinicalTrial.gov NCT00872430

## Background

The phytotherapic product assessed in the present study contains fruits of *Pimpinella anisum *L. (green anises), fruits of *Foeniculum vulgare *Miller (fennel), flowers of *Sambucus nigra *L. (sabugueiro), and flowers of *Cassia augustifolia *(senna plant). Although this combination has been commercially available in Brazil since 1926 [1], its efficacy and safety has never been assessed in a randomized clinical trial.

*Pimpinella anisum *has as chemical representative, namely the anise oil (1-4%). The major component of anise oil, trans-anethole (75-90%), is responsible for its characteristic taste and smell, as well as for its medicinal properties [2,3]. Other constituents include coumarins (umbelliferone, umbelliprenine, bergapten, and scopoletin), lipids (fatty acids, beta-amyrin, stigmasterol and its salts), flavonoids (flavonol, flavone, glycosides, rutin, isoorientin, and isovitexin), protein and carbohydrate [2]. Anise is well known as a carminative and an expectorant, and it is also used to decrease bloating, especially in pediatric patients. At higher doses, it is used as an antispasmodic and antiseptic [2,3], and in vitro studies have also reported on antimicrobial properties of anise [2].

*Foeniculum vulgare *contains essential oils (2-6%) composed of up to 50-70% of trans-anethole and up to 20% of fenchona, in addition to small amounts of limonene, camphor, and alfa-pinene. The fruits and leaves of *Foeniculum vulgare *have been shown to contain a number of flavonoids (quercetin, isoquercetin, kaempferol 3-glucuronide, and kaempferol 3-arabinoside), fixed oil, protein, and organic acids [2,3]. Fennel is used as a laxative in the treatment of mild digestive disorders due to its gastrointestinal effects, namely stimulation of motility and, at higher concentrations, antispasmodic action [4]. Administration of fennel seed oil emulsion has been shown to be superior to placebo in reducing the intensity of infantile colic in a randomized trial [5].

*Sambucus nigra *contains essential oils, free fatty acids (palmitic acid), flavonoids and their glycosides (rutin, isoquercitrin, hyperoside and quercitrin), chlorogenic acid, tannins, and mucilage. It is often used for its laxative effects [3].

*Cassia augustifolia *contains anthraquinones (dianthrone glycosides and sennosides), carbohydrates (polysaccharides and mucilage), flavonoids (isorhamnetin and kaempferol), glycosides (6-hydroxymusizin and tinnevellin), and other constituents. Senna is a potent laxative, and its use in patients with chronic constipation has been assessed. However, an increased number of adverse effects, mainly abdominal pain, have been associated with senna when compared with other laxatives [2-4].

In spite of the beneficial effects shown *in vitro *and *in vivo *for each of the product components described above, the effectiveness of the compound has never been assessed in a randomized clinical trial. In an animal toxicity test, it was considered *innocuous *in rats and rabbits even at a dosage 10 times higher than that used in humans [6].

As an alternative treatment option, phytotherapy may offer advantages in terms of safety, tolerability, and costs, improving patient compliance especially in chronic disorders and long-term treatments [7]. A systematic review of traditional medical therapies for chronic constipation concluded that there was paucity of data regarding commonly used agents such as senna [8]. Another systematic review carried out in the Cochrane Library on the treatment of constipation in palliative care patients found inadequate experimental evidence and insufficient data from randomized controlled trials [9].

Following guidelines of the World Health Organization on the safety of herbal medicines, the Brazilian Health Surveillance Agency requires that phytotherapic products be submitted to efficacy and safety evaluation. In this sense, a randomized clinical trial would provide reliable data for evidence-based decision making and thus increase the acceptance of phytotherapy among physicians [10].

Therefore, the main objective of the present study was to evaluate the laxative efficacy of the phytotherapic compound by objective measurement of colonic transit time (CTT) and clinical variables (number of evacuations per day and perception of bowel function). Secondary endpoints were the evaluation of safety (adverse effects) and quality of life.

## Methods

### Subjects

The target population of the present study comprised patients with chronic constipation aged 18 to 50 years. The following criteria were taken into consideration for the diagnosis of chronic constipation, as defined by the American Gastroenterology Association (AGA): no criteria for irritable bowel syndrome, no loose stools, and at least 12 weeks presenting the following conditions (two or more) in the preceding 12 months: (i) straining in > 1/4 defecations; (ii) lumpy or hard stool in > 1/4 defecations; (iii) sensation of incomplete evacuation in > 1/4 defecations; (iv) sensation of anorectal obstruction/blockage in > 1/4 defecations; (v) manual maneuvers to facilitate defecation in > 1/4 defecations; (vi) < 3 defecations per week [11,12]. Other inclusion criteria were correct filling of a constipation questionnaire during the selection phase and normal laboratory measurements (complete blood count, serum creatinine and potassium, fasting plasma glucose, and thyroid-stimulating hormone). Women in fertile age should be making use of appropriate anticonception. All selected patients signed a written informed consent form.

The following exclusion criteria were also considered: current use of medications with known constipating effects (such as opiates, calcium channel blockers, tricyclic antidepressants, and anticholinergic drugs), pregnancy or breastfeeding, history of alcohol or drug abuse, and any other significant or non-controlled disease.

This study complied with good clinical practice standards, and the study protocol was reviewed and approved by the Scientific and Ethics Committee of Hospital de Clínicas de Porto Alegre at Universidade Federal do Rio Grande do Sul, Porto Alegre, Brazil.

### Crossover and washout periods

This was a randomized, placebo-controlled, single-blinded, crossover study. The order of treatments was counterbalanced across subjects: half of the subjects received the phytotherapic compound for a 5-day period, whereas the other half received placebo for the same period. Both treatment periods were separated by a 9-day washout period followed by the reverse treatment for another 5-day period. In the washout period, patients were free to use any other laxative.

### Procedures and data collection

Clinical evaluation, i.e., clinical history and physical examination, was performed in the beginning and end of each 5-day period. Laboratory evaluation was carried out in the beginning of the study and in the end of each study period. Radiological exams with radiopaque markers were performed in the end of each phase (placebo and active tea) to evaluate CTT. The radiologist was blinded to the intervention.

During the two phases of the study, a constipation questionnaire assessing intestinal transit, straining, stool consistency and sensation of fecal obstruction or incomplete evacuation in the last 24 hours was filled by patients on a daily basis. Since there is no validated Portuguese version of a questionnaire for constipated patients, a new questionnaire was created with nine questions based on the AGA criteria and the Bristol Stool Form Scale [13] (Table 1). We also included a question to assess the patient's own perception of bowel function, in a scale ranging from 1 (very poor) to 5 (excellent). In the beginning and end of each phase, quality of life was evaluated using the WHOQOL-Bref [14], since no specific instrument for quality of life evaluation in constipation has been validated in Brazilian Portuguese.

**Table 1:** Constipation questionnaire and baseline characteristics (n = 20; 19 female)

Question	Answer	(%)
Q1. Have you evacuated in the last 24 hours?	Yes	45
	No	55
Q2. What is the usual consistency of your feces?*	Type 1-3	55
	Type 4	15
	Type 5-7	25
	Missing	5
Q3. Have you made any straining when trying to evacuate in the last 24 hours?	Yes	75
	No	20
	Missing	5
Q4. What was the level of straining in the last 24 hours?^†^	Severe	38
	Moderate	44
	Mild	13
	Missing	6
Q5. Do you have any history of anorectal obstruction?	Yes	15
	No	85
Q6. Do you have any history of incomplete evacuation?	Yes	95
	No	5
Q7. Do you have any history of abdominal discomfort?	Yes	100
Q8. Do you think you have spent too much time trying to evacuate in the last 24 hours?	Yes	20
	No	80
Q9. How do you define your bowel function?	Very bad	20
	Bad	65
	More or less	10
	Good	5

* Bristol Stool Form Scale. ^† ^Not applicable for 4 patients (Q3 = No) (n = 16).

### Characterization of study substances

According to manufacturer instructions, medicinal plants *Pimpinella anisum*, *Foeniculum vulgare*, and *Sambucus nigra *were dried at room temperature and grinded separately by a Weg MOI-02 hammer mill. *Cassia augustifolia*, on the other hand, was submitted to an 8-hour dehydration process in a recirculation stove at 90°C before being grinded. Then, the medicinal plants were homogeneously blended in the following proportion: 2.0 g of *Pimpinella anisum *fruit (green anises), 2.0 g of *Foeniculum vulgare *fruit (fennel), 5.0 g of *Sambucus nigra *flower (sabugueiro), and 6.0 g of *Cassia augustifolia *flower (senna) to every 15 g of blend. The resulting tea was finely packed by Laboratórios Klein (Porto Alegre, Brazil, 2005).

Chemical characterization of the tea was made using high-performance liquid chromatography (HPLC-UV). Analysis of the ethanolic extract (80%) was performed on a Waters 2695 liquid chromatograph equipped with an autosampler and a Waters UV/vis detector model 2675 controlled by the Empower software (Waters, Milford, USA). The column was an RP-18 Nova-Pak^® ^150 × 3.9 mm i.d., 4 μm particle diameter (Waters, Milford, USA). A LiChrospher pre-column (10 × 4 mm i.d.) packed with Bondapak C18 10 μm (Waters, Milford, USA) was employed. Separation was carried out using a mobile phase with water: phosphoric acid (100:0.08, v/v) as solvent A and acetonitile (100) as solvent B at a flow rate of 0.6 mL/min. The gradient program was 90-10 B (0 min), 77-23 B (18 min), 0-100 B (25 min), and 0-100 B (30 min). The system was maintained in equilibrium for 5 minutes with solvent A before the next injection. The injected volume was 10 μl. Chromatographic peaks were detected at 256 nm by comparing the retention time with authentic reference samples of rutin and sennosides A and B. Chromatographic separation was achieved at room temperature.

### Interventions

The phytotherapic product was supplied by the manufacturer (Laboratórios Klein, Porto Alegre, Brazil) as a homogeneous mixture of dried botanicals.

Tea was prepared using 15 g of the compound infused for 5 minutes in 1,500 mL of boiling water. Patients received 150 mL of active tea (equivalent to 1 g of the phytotherapic product) or placebo tea three times a day for 5 days. In order to assure adherence to the study intervention, patient selection was restricted to employees of the hospital where the study was developed. Patients were asked to come to the research unit three times a day to regularly take the tea in front of the investigator. The pharmacist prepared and administered the tea three times a day during the trial so that all patients could receive fresh tea.

### Randomization

Patients were randomized to the treatment or the placebo arm using a single computer-generated random list of numbers. Only the pharmacist was not blinded to patient allocation.

### Blinding

The research pharmacist in charge of preparing and administering both teas (active and placebo) did not participate in data collection and did not have any other interaction with patients and investigators. In order to produce a placebo tea with similar taste and color, 7 drops of caramel color and 10 drops of orange essence were added to 1,600 mL of boiling water. Patients might have recognized which tea was placebo and which was active tea; for this reason, we conservatively considered this trial to be single-blinded. However, both the clinician and the radiologist who measured the primary endpoint and applied the questionnaire were blinded to the intervention during the entire trial.

### Outcomes

Primary outcome was CTT, measured through a radiological technique in which patients were given capsules prepared by a pharmacist, containing 20 radiopaque markers, for three consecutive days. Forty eight hours after ingestion of the last capsule (day 5 and day 19), an abdominal X ray was performed and the number of markers still present in the colon was counted. For determination of the CTT (measured in hours), the following formula was employed: CTT = 1.2 × NMXr, where NMXr is the number of radiological markers found in the abdominal X ray and 1.2 is a constant used by Metcalf et al. [15].

Secondary endpoints were daily symptoms as informed in the constipation questionnaire, quality of life, and incidence of adverse events. Any new symptoms or physical/laboratory abnormalities potentially related with the use of the product were considered as adverse effects by the assisting doctor. The use of other laxatives during the washout period was also measured as a secondary outcome.

### Sample size calculation

Sample size was calculated by estimating that a 20-hour difference in mean CTT would be found between placebo and active tea at the end of the study. Using data from a Brazilian sample of constipated patients were mean CTT (± standard deviation) was 113.1 ± 23.1 hours [16], with a power (1-β) of 80% and an alpha error of 0.05, the minimum number of patients required to detect a statistically significant difference was 22 in each period.

### Statistical analysis

All analyses were based on intention to treat. Fisher's exact test was used for the comparison of rates and proportions. A paired sample *t *test was used for continuous variables at a one-point comparison. For repeated measures, linear regression analysis was used for intra- and inter-group comparisons. Wilcoxon signed-rank test and Friedman test were used to compare the number of evacuations per day (measured as intervals). Statistical significance was set at 5%.

### Study termination

An interim analysis determined *a priori*, performed with 20 patients (40 measurements) by an independent investigator, revealed the presence of statistically significant results, thus leading to early termination of the study.

## Results

A total of 20 patients fulfilled the inclusion criteria. Baseline characteristics of the sample are shown in Tables 1 and 2. Clinical variables such as arterial blood pressure, heart frequency, and laboratory data did not show significant changes during the study. Results obtained in the constipation questionnaire applied at baseline are shown in Table 1. Given the cross-over design of the study, each patient served as his/her own control, thus reducing the influence of variables such as physical activity, body mass index or dietary habits upon the outcome.

**Table 2:** Age, blood pressure, body mass index (BMI), and laboratory assessment of patients (n = 20; 19 female)

Data	Mean	**Min**.	**Max**.	SD
Age	38.9	25	54	7.49
Systolic blood pressure (mmHg)	120.8	96	140	13.89
Diastolic blood pressure (mmHg)	77.2	60	100	9.72
BMI (kg/m^2^)	26.1	18.8	39	4.5
Thyroid-stimulating hormone (μUI/mL)	1.49	0.50	2.80	0.65
Serum creatinine (mg/dL)	0.68	0.5	1.0	0.12
Fasting glucose (mg/dL)	91	78	99	5.80
Serum potassium (mEq/L)	4.50	4.0	4.9	0.25

### Efficacy analysis

**Figure 1:**
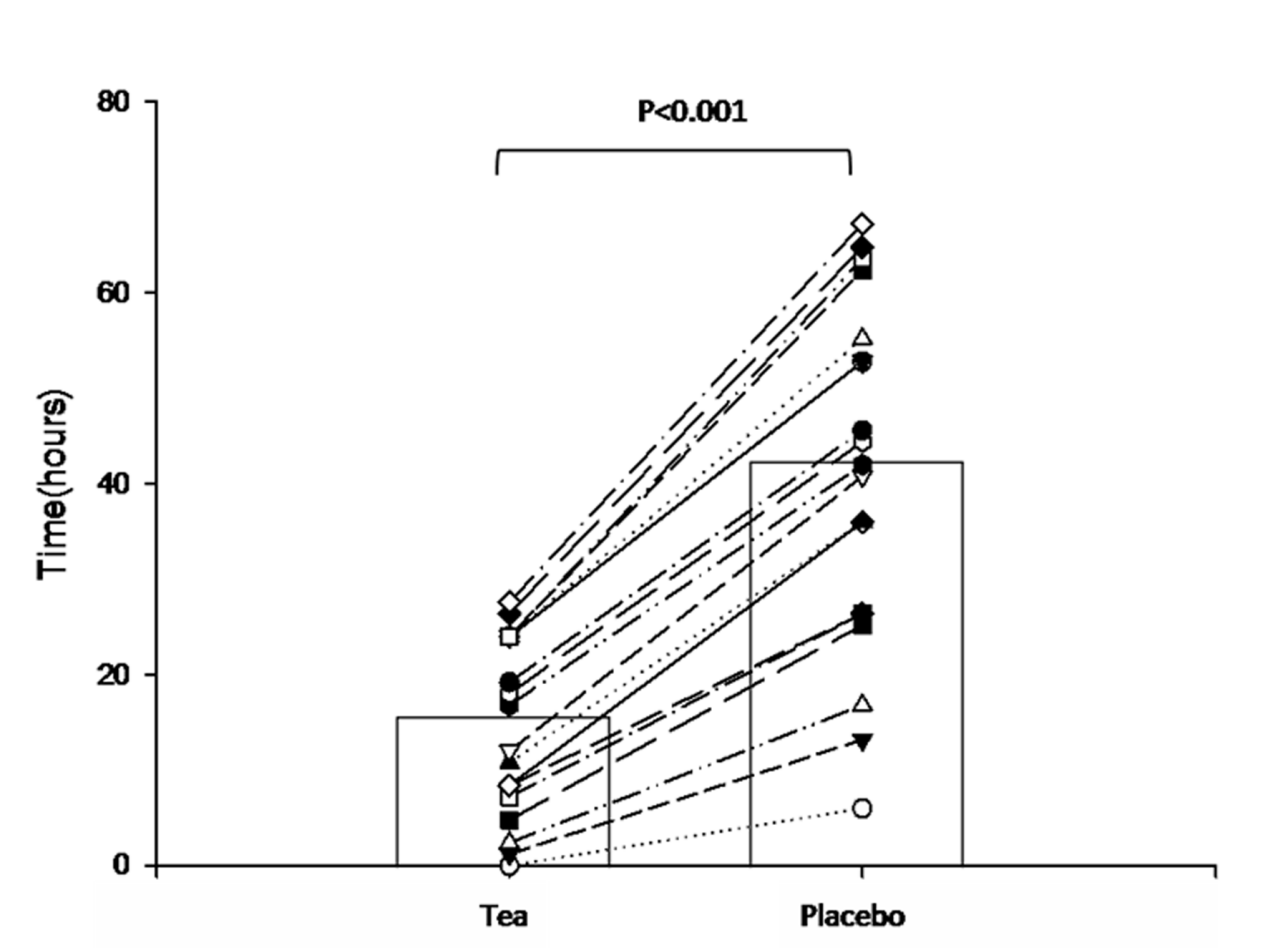
**Colonic transit time according to treatment periods assessed by X ray**. Points represent individual patient values and bars represent mean colonic transit times (in hours). Mean difference between active tea and placebo: 26.6 hours (95%CI 18.7-34.6) favoring active tea.

Mean CTT assessed by X ray was 15.7 hours (95%CI 11.1-20.2) and 42.3 hours (95%CI 33.5-51.1) (p < 0.001) at the end of the treatment periods with active and placebo teas, respectively (Figure 1). The mean difference of 26.6 hours (95%CI 18.7-34.6) between active tea and placebo represented a 62.9% improvement in CTT favoring the active tea during the 5-day assessment period, well beyond the expected during sample size calculation. The magnitude of this difference determined early study termination after an interim analysis.

**Figure 2:**
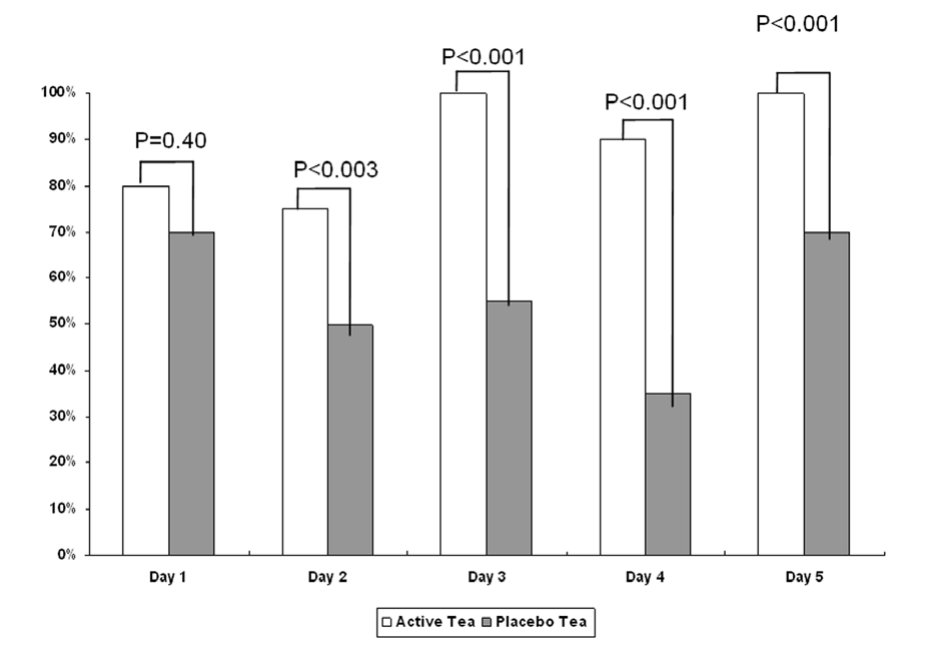
**Proportion of one or more evacuations/day according to treatment period (active tea vs. placebo)**.

As to the subjective analysis, particularly of note was the question "How many times have you evacuated in the last 24 hours?": during the placebo treatment, 42% of the patients answered "none," whereas during the active treatment, only 14% gave the same answer (Fisher's exact test, p < 0.001). A comparison of the number of evacuations per day during the study period as informed in the questionnaire revealed significant differences among groups as of the second day of treatment (Wilcoxon signed-rank test) (Figure 2).

**Figure 3:**
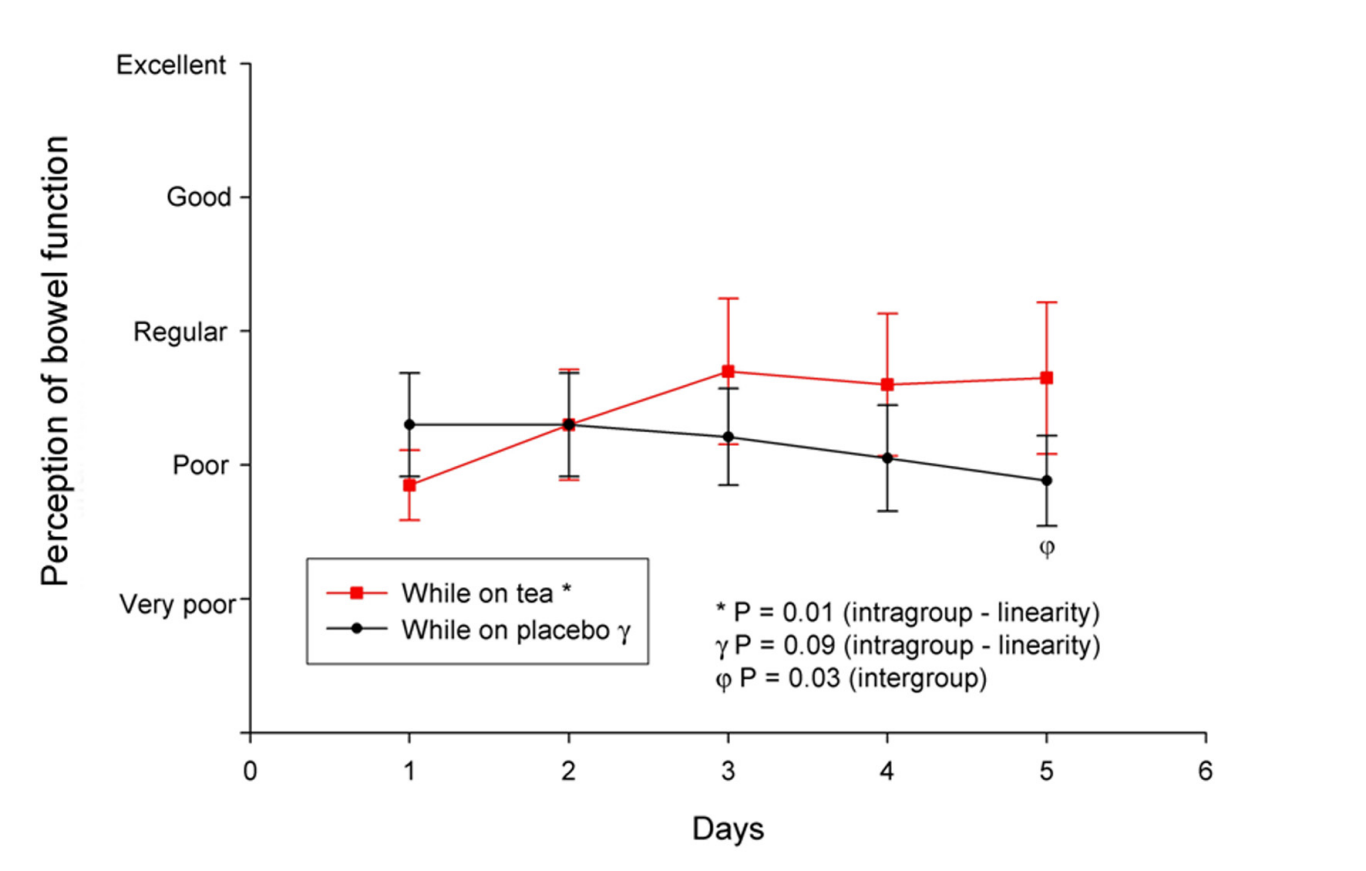
**Bowel function perception by patients according to treatment period (active tea or placebo)**. Values presented as means ± standard deviation.

The question regarding perception of bowel function revealed a significant improvement in the active treatment period (intragroup linearity test, p = 0.01). During the placebo period, on the other hand, there were no significant changes in that classification (intragroup linearity test, p = 0.09). At the end of the fifth day of treatment, a small difference among the groups could be observed (paired sample *t *test, p = 0.03) (Figure 3).

During the washout period, four patients reported the use of laxatives after receiving placebo, and five patients after receiving the active tea. No significant difference was detected in the use of laxatives during the washout period (Fisher's exact test, p = 1.00).

### Safety analysis

Quality of life, evaluated with the WHOQOL-Bref questionnaire, did not show significant differences between the two study periods (*t *test, p = 0.2). A small reduction in mean heart rate was observed during the active treatment period (from 78 in the placebo phase to 70 in the tea phase; p < 0.001), a difference that may be explained by stimulation of the vagus nerve as a result of intestinal cramps.

**Figure 4:**
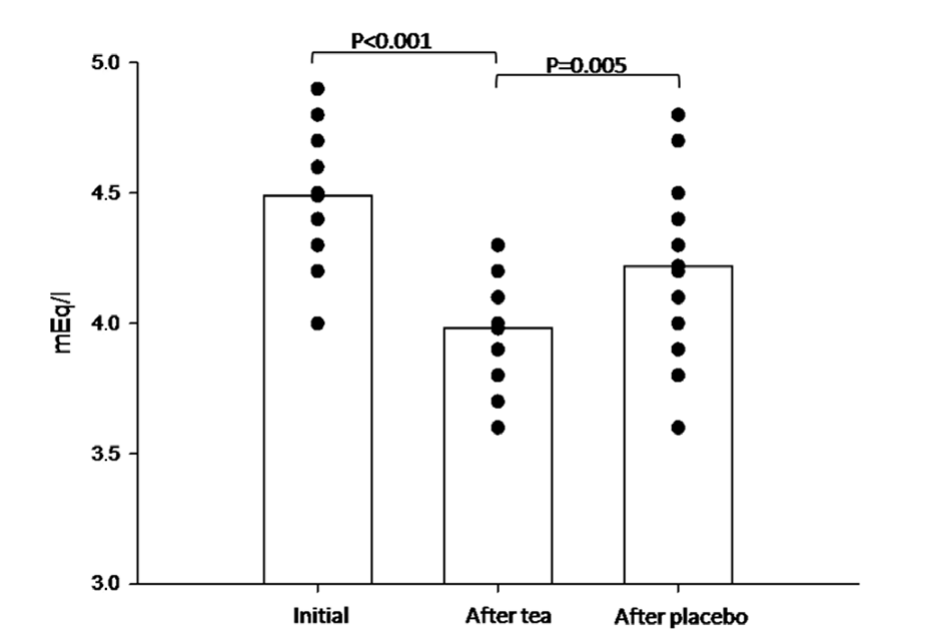
**Serum potassium: individual data and mean values according to treatment period**.

Some laboratory exams showed statistically significant differences between the groups, however of no clinical relevance, once all results remained within normal ranges. The only significant difference detected was a decrease in serum potassium (from 4.50 mEq/L to 3.96 mEq/L; p < 0.001) (Figure 4). No severe adverse effects (e.g. death or risk/disabling events requiring hospitalization or leading to study withdrawal) were observed throughout the study period.

## Discussion

The tea prepared with the phytotherapic compound assessed in the present study presented significant laxative effects when compared with placebo. This effect was demonstrated by a decrease in CTT, as well as an increase in the number of daily evacuations. A few patients also presented diarrhea and colic while on treatment with the active tea.

The addition of herbs such as fennel to this type of compound is a traditional strategy to minimize the cramping caused by senna and *Sambucus nigra*. Fennel may thus be the ingredient responsible for the control of intestinal colic in our patients.

Phytotherapy is a good alternative treatment for constipation as it may offer a few advantages in relation to other treatment approaches, e.g. in terms of safety, costs, and improved patient compliance over a long-term treatment. However, there is currently a paucity of data regarding commonly used agents, such as senna [8]. The present randomized clinical trial was carefully designed and conducted, and therefore we believe it can be used as a reliable source for evidence-based decision making.

In our study, previous use of laxatives was not an exclusion criterion, because it would be very difficult to find treatment-naive constipated patients. Due to ethical reasons, our patients were free to use any other laxative during the washout period. Moreover, the Research and Ethics Committee that approved the study protocol pondered that 14 days (for patients allocated first to placebo) would be too much time without treatment. Fortunately, the use of laxatives during the washout period was not a bias, since no significant differences were observed in the frequency of their use after each of the study periods.

A small decrease in potassium serum levels (within normal ranges) suggests the need for dose titration in patients at risk for hypokalemia, such as users of diuretics. This finding may be explained by the diarrhea experienced by two of the volunteers during treatment with the active phytotherapic tea. Nevertheless, dose titration according to therapeutic effect is probably enough to control this adverse effect.

The radiological investigation of CTT is not regularly adopted in the clinical setting. However, our study showed that this is a very objective way of measuring the final effect of a laxative. The use of clinical information provided by the patient in the research setting is useful but frequently biased by the patient's subjective perception of the treatment. Both methods may have flaws and may not reflect real improvement of colonic function. However, the combination of clinical variables and CTT assessment, as used in this trial, allowed to obtain a more precise and accurate evaluation of bowel function.

No change in quality of life was observed; this may be explained by the short duration of the study and also by the fact that WHOQOL-Bref is a generic instrument for the assessment of quality of life, thus not sensitive enough to evaluate this specific clinical situation.

## Conclusions

This is the first randomized controlled trial to demonstrate the safety and laxative efficacy of a phytotherapic tea containing *Pimpinella anisum*, *Foeniculum vulgare*, *Sambucus nigra*, and *Cassia augustifolia*. Considering the high costs and compliance problems of long-term treatments currently available for chronic diseases such as constipation, this compound comes up as an alternative treatment approach that would be most welcome by doctors and patients.

## Competing interests

The authors declare that they have no competing interests. ATH received a grant from the National Council of Scientific and Technological Development (CNPq) to perform the chemical characterization of the product and to sponsor the clinical trial.

## Authors' contributions

PDP was the principal investigator, conceived the study, and participated in its planning, design, coordination and in drafting the manuscript. RVP reviewed the literature, reviewed the data, helped draft the manuscript, and participated in the statistical analysis. AFC carried out the data collection and helped in data analysis. GBS reviewed the literature and participated in study design and data collection. KMA allocated the patients to the groups, prepared the teas, followed its administration, and helped draft the manuscript. ALA and ATH performed the chemical characterization of the phytotherapic tea using HPLC-UV. All authors read and approved of the final version of the manuscript.

## Pre-publication history

The pre-publication history for this paper can be accessed here:

http://www.biomedcentral.com/1472-6882/10/17/prepub
